# Coproducing Quality and Safety Improvement Projects in Resource-Constrained Countries: Lessons From Mozambique

**DOI:** 10.3389/ijph.2025.1607847

**Published:** 2025-03-12

**Authors:** Paulo Sousa, Edite Thuzine, David Weakliam, Joana Maia, Elenia Amado, Thora Burgess, Peter Lachman

**Affiliations:** ^1^ NOVA National School of Public Health, Public Health Research Centre, Comprehensive Health Research Center (CHRC), NOVA University Lisbon, Lisboa, Portugal; ^2^ Ministry of Health, Maputo, Mozambique; ^3^ Global Health Programme, Health Service Executive, Dublin, Ireland; ^4^ Health Unit, Primary Healthcare Cluster Estuário do Tejo, Lisbon, Lisboa, Portugal; ^5^ Department of Health Systems Policy and Management, National School of Public Health, New University of Lisbon, Lisbon, Lisboa, Portugal

**Keywords:** improvement science, coproduction, quality and safety, maternal & child health, resource constrained countries

## Abstract

**Objectives:**

Mozambique is a large country with low GDP and dispersed population. The health service has limited human and physical resources. These constraints have the potential to result in poor quality of care with an impact on patient safety and person experience.

**Methods:**

This paper is a “before and after” assessment of a quality and safety improvement project based on a qualitative and quantitative review.

**Results:**

Four case studies illustrate the success of the programme with gains in terms of reduction of maternal death and Key lessons are that aid agencies need to coproduce solutions with the local MoH and clinical teams so that there is ownership of the programme. Thus, all interventions need to be financially light, i.e., aiming to achieve success with minimal funding, so that when the programme ends there is a sustainable plan that can be maintained.

**Conclusion:**

In this review of quality improvement initiatives in Mozambican hospitals we have demonstrated the potential to enhance patient outcomes despite resource constraints. The key to the success of the initiative has been collaborative work as equal partners.

## Introduction

Universal Health Coverage (UHC) is the goal of many health systems worldwide. The World Health Organization (WHO) has emphasised that UHC healthcare must be safe and of a high-quality [1, 2]. Therefore quality improvement and patient safety methodologies are critical for the effective implementation of UHC [1]. Low-income countries (LIC) such as Mozambique have the additional challenge of limited resources and infrastructure, which impede the implementation of UHC.

The aim of this paper is to demonstrate how to coproduce improvement initiatives in a resource poor environment by developing a partnership between an LMIC MOH, a donor organisation, and frontline teams so that there is effective and sustainable improvement in delivery of care The paper provides an overview of an innovative programme aimed at strengthening hospitals in the local healthcare system, so that they are able to deliver high quality and safe person-centred care through a technical partnership with the Irish Health Service Global Health (HSEGH) programme. We explore the transformative power of local leadership and skills at both central and hospital levels to deliver quality and safety with catalytic support by the international collaborator. The innovation is on how resource and infrastructural limitations can be overcome by coproducing better use of existing resources, drawing on lessons from successful and unsuccessful interventions.

### Context

Mozambique is one of the largest countries in Africa with the capital Maputo close to South Africa in the south, while the northern border with Tanzania is 2,300 km away. The population is mainly rural and dispersed, resulting in challenges for the delivery of equitable high-quality care across the country. As in many countries, there are competing healthcare goals requiring the careful allocation of limited resources. Mozambique was ranked 185 on the Human Development Index in 2021 [3]. The country has many health challenges that need to be addressed with limited resources [4].

In many Lower (LIC) and Lower Middle-Income Countries (LMIC) the challenges for people are similar, though contexts may be different [5]. The provision of quality and safety in patient care is difficult, as there are challenges in infrastructure, supply chains and personnel. These constraints have the potential to result in poor quality of care with an impact on patient safety and person experience. Maternal and child health, surgical complications, deterioration, infections and medication safety predominate, as well as delays in access to care [6, 7]. While UHC aims to enhance access, it must be coupled with a parallel focus on addressing the quality of care [8]. Therefore, it is essential to develop innovative and cost-effective initiatives to ensure high quality care within the context of UHC [9, 10].

#### The Strategic Partnership to Improve the Quality and Safety of Care

In 2014, during an official visit to Ireland by the President of Mozambique, the Irish Health Service Executive (HSE) signed an agreement with the Ministry of Health (MoH) in Mozambique to collaborate to improve health services. The agreed overall aim was to build local capacity to address the challenges of quality and safety. The underlying principle of the agreement was one of coproduction and codesign of the interventions in an equal and reciprocal partnership.

Over the next year the MoH decided to prioritise the issue of poor quality of care at hospitals. The programme was co-designed by the Directorate for Medical Services with the HSEGH, while the Irish Embassy in Maputo provided logistic and financial support for capacity building workshops. A technical advisor from the International Society for Quality in Healthcare (ISQua) provided improvement and patient safety science expertise.

A key element of the programme has been to raise the profile of quality in healthcare at ministerial level. Prior to the agreement the Minister of health would not have been involved in quality and patient safety initiatives and a dedicated quality department did not exist in the MOH. In the design of the progamme it was recognized from the start that leadership at the highest level was essential to ensure success of the initiative. The Irish Embassy facilitated meetings with the Minster of Health and directors of departments, thereby laying the groundwork for the success of the programme from a policy and strategic perspective.

#### The Context of the Intervention 2016–2023

The improvement intervention was based on a modified Breakthrough Series Collaborative [11]. From 2016 to 2018 two to three workshops were held a year and in the intervening periods the MoH provided support to monitor and supervise the teams in the implementation of their projects.

The MoH selected teams from 14 hospitals across Maputo and five provinces. The selection of hospitals was one of convenience and where the MOH considered the hospitals ready to enter into an improvement programme. Therefore there were more hospitals from the Maputo region for convenience and selected hospitals from the other provinces.

The team members included medical and nursing leaders as well as other healthcare professionals pertinent to the problem being addressed, e.g., pharmacists, physiotherapists and administrators/managers. The MOH identified healthcare workers with potential leadership skills who could champion initiatives to improve quality and safety at their hospital as quality improvement (QI) trainers. Improvement Science methodology was introduced and adapted for the local context. In 2017 each team was given a copy of the Portuguese version of The Improvement Guide [12]. In 2018 the MoH translated the Aurum Institute’s How to Guide for Quality Improvement [13] into Portuguese and electronic copies were distributed to teams in the programme.

Initially, the MoH wanted to run a collaborative on a specific topic. The technical team recommended that it would be more appropriate if the teams decided what mattered to them. The teams identified problems that they faced (Box 1). This allowed coproduction of solutions that were relevant to their context.

BOX 1 **Problems Identified by clinical teams.**

Teams identified problems in different way depending on their context. These included adverse events, complaints from patients and families, staff experience of service delivery, waiting times and lists.

• High mortality within 24 h of admission to medical wards.
• High rates of complications following surgery.
• Inadequate medical records.
• Prescribing errors.
• Patients absconding from a mental health facility.
• Delays in the Emergency Department.
• Delays in Outpatient clinics in HIV Services.
• Delays in gynaecology clinics.
• Poor communication in HIV clinics.
• Issues in transfer from rural hospitals to tertiary care.
• Maternal and child health.

All of the identified issues and the initiatives that followed were representative of the different domains of quality, with safety and access to timely care being predominant. All learning sessions in Mozambique were facilitated with simultaneous translation into Portuguese. The programme converted to virtual in 2020 and 2021 due to the COVID-19 pandemic. This added to the complexity of language differences and the HSEGH invited the NOVA National School of Public Health in Lisbon to be part of the technical team to provide support in Portuguese. In person sessions recommenced in 2022 and a hybrid delivery model is currently used for knowledge transfer. A further series of workshops were conducted in 2022 and 2023 with new teams identified from 14 hospitals across the country.

## Methods

In global health development initiatives, a “before and after” assessment of progress of the intervention does not reveal the richness of the experiential changes or of some clinical improvements [14]. Therefore, this paper is a retrospective narrative, qualitative and quantitative review of the programme. In the review we provide narrative feedback on the retrospective analysis of the healthcare improvement initiatives undertaken in Mozambique in collaboration with the HSEGH from 2016 to 2023. Four case studies illustrate the progress made to date.

The workshops were designed by the technical team and were based on the theories of Deming, Juran and Shewhart for quality improvement. For patient safety the theories of reliability, human factors and ergonomics (HFE) and resilience engineering, as well as managing risk were included. All workshops were participatory including a blend of didactic teaching and team interaction.

The hospitals chosen to illustrate the success of the programme were selected to provide breadth in problems solved and to illustrate different outcomes. Where relevant they were required to have data to demonstrate progress in their projects.

Depending on the type of initiative, data on patient outcomes, care processes, and patient satisfaction have been collected from hospitals participating in the QI projects over a span of two distinct phases. The first phase covered the period from 2016 to 2020, and the second from 2021 to 2023.

## Results

The QI programme has had an impact on the Ministry of Health as well as individual hospitals. A key achievement has been the facilitation of opportunities for knowledge exchange and sharing of best practice. This has focused on developing sustainable change to allow for continuous improvement. The MoH has been critical to promoting nationwide QI efforts to ensure the long-term viability of projects beyond the institutions that have participated in the programme. The programme has raised the profile of quality and safety in the MOH and as a result in 2017 the MoH established a Directorate for Quality and Standards, with the remit of facilitating regular monitoring and coaching sessions with hospitals, undertaking national QI projects, and spreading successful initiatives. Hospitals have been supported by the MoH to address immediate challenges and to develop strategies for the future.

To illustrate the impact of the programme, four case studies are provided. These have been selected to demonstrate success, facilitators, barriers and challenges faced by the teams and are representative of the other teams in the programme.

In Box 2 a patient safety initiative to identify the risk of deterioration of newly admitted patients to an acute medical ward is described.

BOX 2 **Deterioration of patients at Jose Macamo General Hospital in Maputo.**

**FIGURE 1:**
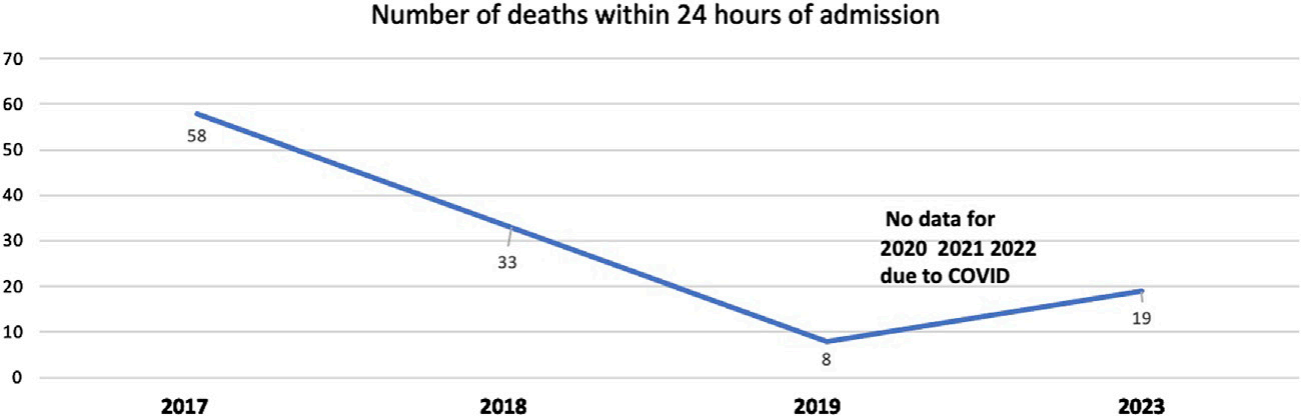
Trends mortality within 24 h of admission, Jose Macamo General Hospital in Maputo (Maputo, Mozambique, 2024).

At Jose Macamo General Hospital in Maputo, the team reported that they had high mortality within 24 h of admission for patients admitted to the acute medical ward. The context was a 75 bedded ward with very few nursing staff. The team were trained in concepts of patient safety with a focus on situation awareness and proactive detection of deterioration. An early warning system to detect deterioration was introduced. It was decided to use the tool “Between the Flags,” a “track and trigger” system to detect deterioration, from the Clinical Excellence Commission in New South Wales Australia [15]. The tool was translated locally, and 5000 copies printed in Dublin as colour printing in Maputo was not feasible. The clinical staff were trained in the use of the tool, and how to predict who may deteriorate. This has resulted in a substantial decline in numbers of patients dying within 24 h of admission from 58/year in 2017 to 8/year in 2019. The data during the COVID pandemic is not available (Figure 1).

Despite COVID-19, the increased pressure on the system, changing staff, moving the ward to a new location, and the need to improvise using greyscale versions once the supply ran out, the gains have been maintained and the number of deaths in the first 10 months of 2023 is lower than it was in 2017, but more than in 2019.

A main focus of the current programme is to address the high rates of maternal death in childbirth across Mozambique. The main causes appear to be failure to manage eclampsia and *postpartum* haemorrhage. Five hospitals from across the country have initiated programmes to improve the identification of women at risk of both conditions and then taking mitigating actions. The initial intervention has been to increase the correct use of the partogram which will allow for earlier identification of risk [16]. In Box 3 an example of one of the initiatives on improving maternal wellbeing is described.

BOX 3 **Improving safety in maternal care at Manica District Hospital.**

Remote provincial hospitals have experienced challenges implementing and sustaining the QI initiatives, due to logistical and resource limitations. The team at Manica District Hospital identified maternal morbidity and mortality as the issue to be addressed in their QI initiative. Although they have relatively few deaths – (three over the past 3 years) - they have experienced high levels of eclampsia and *postpartum* haemorrhage. The team faced challenges in executing their project aimed at reducing eclampsia and *postpartum* haemorrhage. In the assessment of the challenges, they postulated that it was due to the variable quality of antenatal care not facilitating early detection of risk factors, the absence of adequate equipment to monitor women in antenatal care and then in labour, and the lack of trained staff. To address these limitations, the team has initiated a series of workshops to train healthcare professionals in blood pressure monitoring during maternal consultations and to identify women at risk of eclampsia and PPH by improving the use of the partogram. The underlying safety theory was one of reliability, with the aim of improving reliability in the process from a near zero 100% filled out partogram as recommended by the WHO to one where all steps were completed.

**FIGURE 2:**
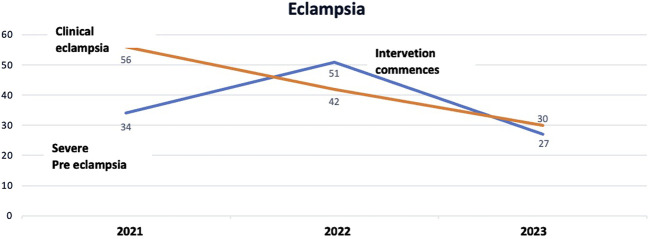
Trends in presentation of eclampsia and pre-eclampsia in 2021, 2022 and from January to October 2023, Manica District Hospital (Manica, Mozambique, 2024).

**FIGURE 3:**
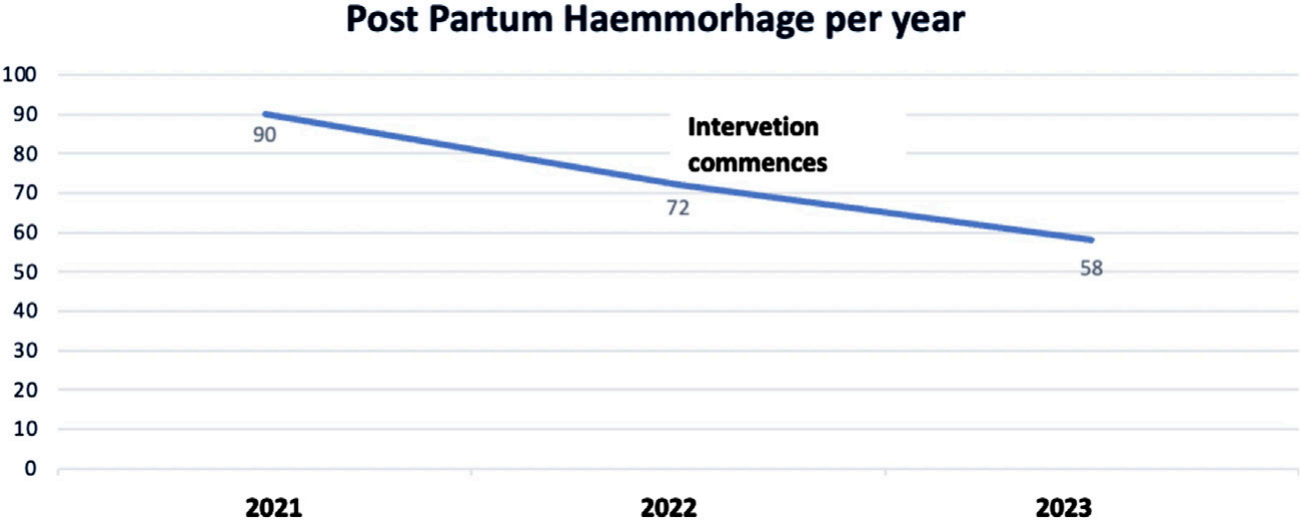
Trends in presentation of *Post partum* Hemorrhage, Manica District Hospital (Manica, Mozambique, 2024).

In Figures 2, 3 the improvements over the past 3 years are demonstrated.

The project commenced at the end of 2022 so the 2023 figures are starting to demonstrate decreased levels of both complications within the context of increasing births from 1797 in 2021, 1903 in 2022 and a projected 2231 in 2023, based on the numbers from January to October. The proactive approach aims to improve patient outcomes and to cultivate a culture of continuous learning and adaptation. However, the success of this initiative also underscores the pressing need for adequate equipment and resources. The team at Manica District Hospital has demonstrated that it is possible to make changes and achieve improvement despite resource limitations.

The focus generally has been on safety and access to care. Nonetheless, person centred care remains a focus for all of the teams. At Beira Central Hospital this has been the main interest from the start, as demonstrated in Box 4.

BOX 4 **Patient-Centred care Initiatives at Beira Central Hospital.**

Beira Central Hospital has had a focus on person-centred care from the first collaborative to the most recent. In 2017, the team identified that patients receiving HIV feedback following diagnosis had a poor understanding of what they were told and expressed dissatisfaction. The Teach-Back method [17] was adapted to the local context and introduced for patients with HIV in the outpatient clinics. This programme experienced the impact of the catastrophic floods that disrupted healthcare services in 2019. In 2022 they recommenced the improvement programme with a project to address the challenge of communication between healthcare professionals and patients, a concern highlighted by patient complaints.

The team has introduced several new initiatives to improve patient care. They prioritized feedback through “Complaint Boxes” and responded promptly to concerns. Patients identified communication to the key area where improvement was required and where this was achieved. Staff training was enhanced with workshops focusing on communication and empathy. Continuity in care was emphasised by announcing doctor changes post-shifts. Furthermore, they have implemented patient satisfaction feedback at discharge to continuously refine their practices.

Sustainability and the spread of the quality improvement methodology has been one of the goals in the programme. QI coaches were trained at each hospital and at some of the hospitals this has resulted in the evolution of one project into system wide projects as demonstrated in Box 5.

BOX 5 **Building sustainability at Mavalane General Hospital.**

**FIGURE 4:**
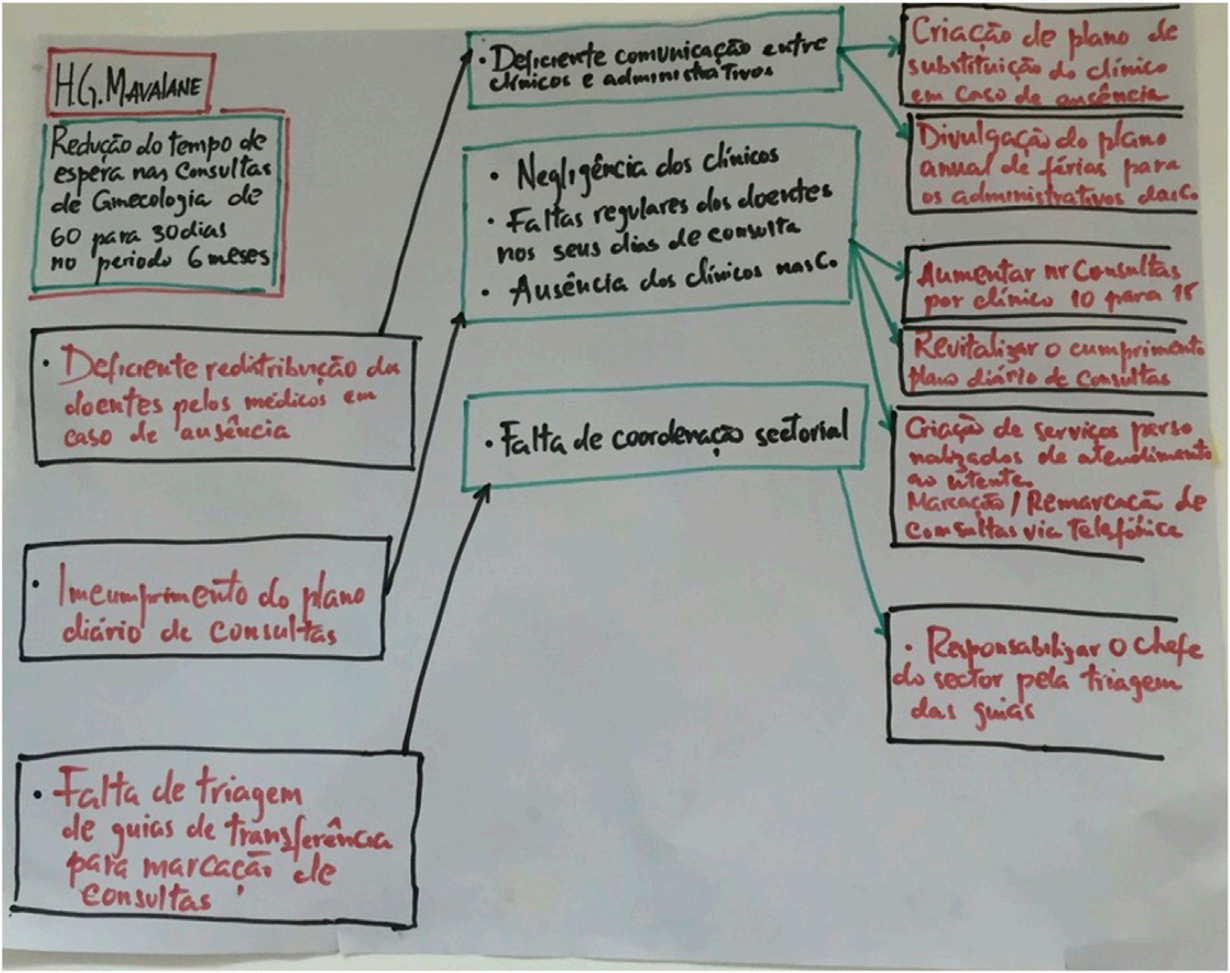
Theory of change–Driver Diagram at Mavalane Hospital (Mavalane, Mozambique, 2024).

At Mavalane General Hospital the first project focused on waiting times for gynaecology outpatients which was over 6 months. The initial project team studied the process and developed a plan based on open access–i.e., a target of providing the first appointment within what was considered to be the shortest time possible, i.e., six weeks. They developed a theory of change as indicated in Figure 4.

The team exceeded the target and were able to offer patients an appointment within 3 weeks of referral. As they recognised this impact the follow up then focused on ensuring that the second appointment was no longer than 3 weeks as well.

The project lead has subsequently been appointed as hospital Clinical Director which has resulted in QI methodology being incorporated into the operational procedures. Mavalane Hospital has systematically integrated the QI methodology across all departments with the objective of optimising patient care. The effectiveness of this integration can be attributed to the hospital’s project champion now being in an executive role, who has undergone continuous training within this programme since 2017. This exemplifies the added value derived from long-term and sustained participation in the programme.

## Discussion

### Statement of Principal Findings

The initiative in Mozambique has differed from other global health programmes in that it was coproduced with the MoH and the clinical teams at the participant hospitals, rather than the technical team just coming in with a solution. The programme operates within a constrained budget, building sustainable change which is not dependent on external finance [18, 19]. Financial and resource limitations have led to unique, resource-optimised solutions. Underlying this approach is the constant attention to the relationship between the MoH and HSEGH. Alignment of local and donor ideas has ensured ongoing success, despite the local challenges and the pandemic.

The QI collaboratives and QI projects have contributed to the development of structures at the central MoH and local levels, to support sustainable quality and safety initiatives. The newly established Directorate of Quality and Standards has supported the introduction of dedicated quality departments in each hospital focussing on site specific projects. The hospital based quality committees work on initiatives to improve patient care so that it is efficient and evidence based.

### Strengths and Limitations

Achieving Improvement is complex with many different factors that facilitate success:

• Leadership within each organisation/hospital has been an essential component of the success of quality initiatives. Local leadership has provided informed, strategic oversight that harmonised the complex interplay of limited resources and increasing demand for services.
• The sustainability of the Quality Improvement methodology has been enhanced by ongoing support from the MoH and the establishment of specialised quality departments in each hospital.
• Provision of Portuguese language knowledge material on the fundamentals of improvement science has minimised the challenge of language. Contextualisation of the theory and methodology ensures that learning has been sustained.
• The addition of a Portuguese speaking technical adviser has enhanced communication and has ensured that the methodology is clearly understood and effectively implemented.
• In-person workshops facilitated the building of relationships between team members and external experts. This has enabled the subsequent virtual learning to have value for the learners.
• The continuity of learning sessions and the interhospital knowledge transfer have been crucial in achieving improvements and ensuring the sustainability of gains.
• Local logistical support has provided the foundation for addressing organizational challenges in the learning sessions.
• The programme is built on the willingness and enthusiasm of learners which promotes a collaborative culture that focusses on problem-solving and continuous improvement.

### Interpretation Within the Context of the Wider Literature

As external facilitators it is important to constantly learn from the success and challenges of working in a low or low- and middle-income country. Important lessons include:

• Coproduction of programmes with those at the front line of service delivery is essential [20, 21]. In many countries the MoH acts in a hierarchical paradigm, issuing top-down directives. As a consequence of this programme real success stories have emerged from the coproduction of the local projects. Balancing the broader strategic objectives with the lived realities of frontline workers, patients, and the community has been an essential component of the programme.
• A long term partnership is fundamental to the success of programmes. The Memorandum of Understanding (MoU) has provided a framework of long term commitment to health service improvement that enhanced the depth of the MoH-HSE partnership. This has emphasised the significance of enduring international alliances. While one-off interventions can make a mark, consistent collaborations bring lasting systemic changes.

Building resilience to deal with unexpected and expected challenges is essential. The local healthcare teams have exhibited remarkable adaptability. They have confronted natural disasters and epidemics as well as resource limitation and have continued to develop new quality initiatives, despite the context of constant uncertainty.

Improving care in low and low-middle income countries has specific challenges that can be dealt with proactively. These include:

• Political factors, including shifting government priorities, acute resource crises, and bureaucratic hurdles.
• Changes in leadership within the MoH departments and structures and local projects sometimes has resulted in brief interruptions of the projects.
• Natural disasters that pose unforeseen setbacks, stretching already limited resources and diverting focus from long-term objectives to immediate crisis management.
• Limited staff capacity at MoH and hospitals which result in an inconsistent schedule for both online and in-person workshops.
• Lack of reliable internet access including poor bandwidth which constrains the virtual learning opportunities.

All of these challenges have required flexibility, revealing the critical need for resilient healthcare systems and hospitals that are capable of maintaining service quality and project momentum, despite external and uncontrolled destabilising factors.

### Implications for Policy, Practice and Research

This programme gives several lessons and interesting insights for decision making process, daily practice and research opportunities in quality and safety improvement in a LIC or LMIC setting.

• Base the initiative on shared values

Establishing a shared set of core values provides a guiding light for any global health initiative. The example from Mozambique suggests that when values are centred around patient care, quality and safety improvement, coproduction and collaboration, they can be a focus for local and international partners.

• Coproduce quality initiatives

Collaborative co-production, as seen between the MoH and HSEGH, leads to a synergistic approach where the strengths of each party are harnessed. The sharing of agency at every level ensures that the programme will be accepted and sustained at local and central levels.

Coproduction ensures that the initiative is tailored to the local context while benefiting from international expertise and perspectives [22]. Ground-level engagement ensures that solutions are realistic, effective, and cater to the local context. Harmonisation of initiatives to the local context is vital [23]. The successes emerging from the local projects in Mozambique underscore the importance of balancing broader objectives with the real-time challenges and needs faced by frontline workers and the community.

• Develop leadership for quality and safety

Leaders must embrace quality improvement as part of their leadership role to enhance the quality of care [24]. Leaders need to provide the time and space for change as this is the foundation of a successful programme. Leadership ensures the necessary direction, aligns disparate efforts, and facilitates the long-term viability of projects by ensuring alignment with broader strategic goals.

• Build relationships to facilitate improvement

Quality of care is more than a technical endeavour and is dependent on relationships [25]. Building relationships during face-to-face interactions creates a stronger bond and mutual understanding among team members and experts. Such engagements, as highlighted in the Mozambican experience, can pave the way for sustained collaboration, clearer communication, and better project outcomes even after the workshops conclude. This facilitates virtual support. Making the interactions fun and respectful strengthens relationships.

• Recognise that patience is a virtue

Initiatives in global health face many challenges, from political shifts to natural disasters. The experience in Mozambique underscores the importance of patience in navigating such challenges. Improvers need to accept the varied pace at which different projects progress, as this will facilitate long-term success. Donor agencies often work to timetables and budget reporting that, most of the time, do not align with the pace of change in the LMIC. It is crucial to remain committed to long-term goals, understanding that change and embedding of improvement takes time [26].

• Engender mutual respect, trust and acceptance of different cultural values

For any international collaboration to flourish, there must be mutual respect and trust between partners. Recognising and acknowledging different cultural values ensures that initiatives are culturally sensitive and relevant. This is essential when working in a post-colonial environment in which donor agencies can easily revert into the colonial construct.

### Conclusion

Enhancing healthcare quality in Africa is still in its early stages, but the prospect for success and substantial health improvements is considerable [27]. In this review of quality improvement initiatives in Mozambican hospitals we have demonstrated the potential to enhance patient outcomes despite resource constraints. The key to the success of the initiative has been collaborative work as equal partners.

This requires co-production within respectful relationships in which the high-income country technical advisors and the LIC/LMIC partners work together to achieve improved outcomes. Partnerships based on these principles can significantly enhance quality improvement and patient safety.

## Data Availability

All data relevant to the study are included in the article.
